# Diffuse large B‐cell lymphoma with phenotype switch, anaplastic features and aberrant T‐cell marker expression during disease evolution

**DOI:** 10.1002/jha2.546

**Published:** 2022-08-21

**Authors:** Xiaoqiong Wang, Pei Lin, Jie Xu, Shaoying Li

**Affiliations:** ^1^ Department of Hematopathology The University of Texas MD Anderson Cancer Center Houston TX USA

**Keywords:** DLBCL, phenotype switch, clonal evolution, anaplastic, aberrant T‐cell marker expression

1

A 41‐year‐old man was referred to our institution for relapsed diffuse large B‐cell lymphoma (DLBCL). He was diagnosed with DLBCL, non‐germinal center B‐cell (non‐GCB) immunophenotype at an outside hospital and received R‐EPOCH for 6 cycles and reached complete remission. He relapsed 3 months later and received RICE (Rituximab, Ifosfamide, Carboplatin and Etoposide) chemotherapy and autologous stem cell transplant for persistent disease, then further received DHAP, and anti‐CD19‐CAR T‐cell therapy combined with ibrutinib. He now progressed with extensive lymphadenopathy and extranodal involvement.

The initial diagnostic biopsy of the cervical lymph node was reviewed and showed a diffuse infiltrate of large lymphoid cells with immunoblastic morphology (Image A‐B). The lymphoma cells were positive for CD20 (Image C), BCL‐6, MUM1, MYC and BCL2, and were negative for CD3 (Image D), CD5 and CD10, diagnostic of DLBCL, non‐GCB type.

In contrast, the current biopsy of the abdominal lymph node showed a diffuse infiltrate of large pleomorphic and blastoid lymphoid cells, with some giant mononucleated or multinucleated cells mimicking megakaryocytes (Image E‐F). The lymphoma cells were positive for CD79a (Image G), PAX5, CD10 (diffuse, Image H), BCL‐6, MUM1, MYC, BCL‐2, CD3 (subset, image I) and CD5 (subset), and negative for TdT and CD61, with a proliferation index of 80% (Image J). Flow cytometry showed aberrant large B‐cells expressing CD5 (partial), CD10, CD20, CD22, and monotypic kappa surface light chain and lacking CD19 (Image K‐L) (Figure 1). Fluorescence in situ hybridization analysis showed no *MYC* rearrangement. The patient was diagnosed as DLBCL, GCB immunophenotype, with anaplastic features and aberrant T‐cell marker expression. He was refractory to treatment and expired within 1 month.

**FIGURE 1:**
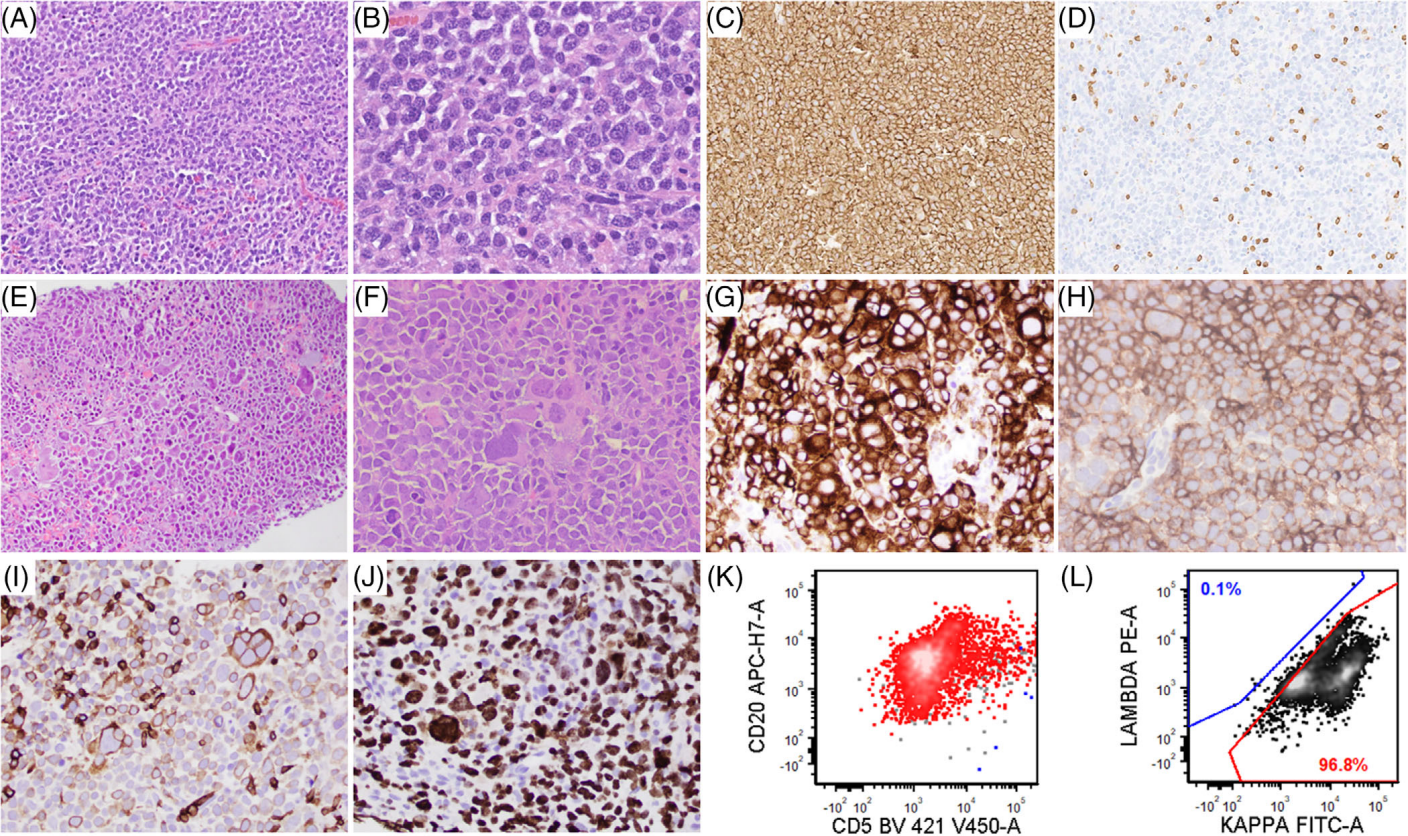
Tissue biopsies at initial presentation (A‐D) and relapse (E‐L). Excisional biopsy at initial presentation showed diffuse infiltrate of large lymphoma cells with immunoblastic morphology (A, x20; B, x40). The lymphoma cells are positive for CD20 (C, x20) and negative for CD3 (D, x20). Core needle biopsy of the relapsed specimen showed diffuse infiltrate of large pleomorphic and blastoid lymphoid cells with giant mononucleated or multinucleated cells mimicking megakaryocytes (E, x20; F, x40). The lymphoma cells are positive for CD79a (G, x40), CD10 (H, x40), and CD3 (I, x40), with a Ki‐67 of 80% (J, x40). Flow cytometry studies show aberrant large B‐cells expressing CD5 (partial), CD20, and monotypic kappa surface light chain (K‐L).

This relapsed DLBCL demonstrated drastic changes in both morphology (from immunoblastic to anaplastic) and immunophenotype (from non‐GCB to GCB), and gained expression of CD10, CD3 and CD5. Recent literature has shown that genetic/epigenetic alterations acquired during the disease evolution drive phenotypic alterations in B‐cell lymphoma, and play a critical role in priming lymphoma cell plasticity and therapeutic resistance. The phenotypic alterations and anaplastic features in this relapsed DLBCL patient indicate therapeutic failure and disease progression, and should be recognized and emphasized to guide appropriate clinical management.

## FUNDING

No funding was required for this study.

## ETHICS STATEMENT

Informed consent was obtained from the patient (“front‐door” consent). PERMISSION TO REPRODUCE MATERIAL FROM OTHER SOURCES.

## CONFLICT OF INTEREST

The authors declare that they have no conflict of interest.

## Data Availability

The data that support the findings of this study are available from the corresponding author upon reasonable request.

